# Preventing Job Burnout: Could Workplace Support Protect Maternal and Child Health Professionals Who Are Doing Public Health Equity Work?

**DOI:** 10.1007/s10995-023-03846-7

**Published:** 2023-11-25

**Authors:** Carol Gilbert, Marilyn Johnson, Bibhusha Karki, Kiara Lyons, Melissa Tibbits, Drissa Toure, Aislinn C. Rookwood, Chad Abresch

**Affiliations:** 1 Department of Health Promotion, College of Public Health, University of Nebraska Medical Center, 984365 Nebraska Medical Center, Omaha, NE 68198-4365 USA; 2 Division of Nursing, Midwifery and Social work, School of Health Sciences, University of Manchester, Manchester, UK; 3 Division of Epidemiology and Community Health, School of Public Health, University of Minnesota, Minneapolis, MN USA; 4 College of Health Solutions, Arizona State University, Phoenix, AZ USA

**Keywords:** Equity, Emotional labor, Job burnout, Workplace support, Employer, Public Health Department

## Abstract

**Purpose:**

To assess the potential of workplace support to protect public health equity workers against job burnout and to identify key workplace support components.

**Description:**

This mixed-methods, explanatory sequential study analyzed survey and interview data collected between August 2020 and June 2021. Participants included governmental and non-governmental public health employees whose programs largely focus on Maternal and Child Health populations and who reported that their jobs involved working to reduce health inequities (“equity work”). Regression analysis tested the effect of emotional labor on job burnout, and whether workplace support modified that effect. Qualitative analysis of interview transcripts explored possible components of needed workplace support.

**Assessment:**

Emotional labor was positively associated with job burnout (p < .001), and there was a significant negative interaction between emotional labor and workplace support, meaning workplace support appeared to reduce the effect of emotional labor on burnout (p = .036). Qualitative analysis identified four support components: peer-to-peer mentoring connections, workplace accommodations, engaged and empathetic supervision, and mental health resources.

**Conclusion:**

Workplace support is associated with reduced job burnout for public health equity workers, especially those whose jobs involve high levels of emotional labor. Few public health employers are providing needed emotional supports for their equity workers, but certain supports appear to be helpful in reducing job burnout.

## Significance

Stressful work environments can impact emotional well-being and lead to job burnout. Since the 1980s, research has explored emotional labor (i.e., self-management of emotions for effective job performance) as a contributor to burnout, including new studies in the healthcare and public health sectors. The recent focus of MCH on equity requires workers to address emotion-laden topics with partners and the public. Past research had not focused on the public health sector and had emphasized personal coping strategies. Our study adds to the literature by examining emotional labor in MCH equity work and considering the protective potential of workplace supports.

## Purpose

According to the 2021 Public Health Workforce Interests and Needs Survey, public health employees have increasing levels of stress, burnout, and intent to leave (de Beaumont Foundation, 2022). For public health professionals working to advance health equity (hereafter, “health equity professionals”), the struggle against structural racism and injustice produces high levels of stress and burnout (Abresch et al., 2022). In recent years, addressing social determinants of health, including racism, has become a core part of the field of Maternal and Child Health (MCH), which serves vulnerable populations in sensitive developmental periods, and often addresses system inequities to achieve health goals (Pies & Kotelchuck, 2014; Rosario et al., 2022).

Research has long demonstrated that stressful work environments can affect mental and physical health and lead to job burnout, defined by Maslach and Jackson as a cluster of physical, emotional, and interactional symptoms correlated to job stress and emotional fatigue (Aung & Tewogbola, 2019; Jeung et al., 2018; Maslach et al., 2001; Maslach & Jackson, 1986). Recently, burnout and its link to job turnover have been studied in the context of public health and healthcare (de Beaumont Foundation, 2017; Lu et al., 2020; Willard-Grace et al., 2019).

One important source of stress in work is emotional labor, which occurs when work requires the management of personal emotions for effective job performance, or to meet workplace norms and goals (Hammonds & Cadge, 2014; Załuski & Makara-Studzińska, 2018). A growing body of research has found high levels of emotional labor and burnout, and a dearth of workplace supports among public health and healthcare workers (Abresch et al., 2022; de Beaumont, 2017; Yeh et al., 2020).

Although personal coping strategies to mitigate the impacts of emotional labor have been extensively investigated, protective systems provided by the workplace (i.e., workplace supports) are comparatively understudied. A handful of studies have shown that supervisors and workplace characteristics can be helpful (Huang et al., 2015; Jeung & Chang, 2021; Whitebird et al., 2013); however, evidence of effectiveness is thin. Thus, research is needed to assess the protective potential of workplace support and identify key components. Our study sought to address this gap with a specific focus on health equity professionals in MCH.

## Description

Two research questions guided the analysis of this study:

- Quantitative: “How is the relationship between emotional labor and job burnout affected by workplace support for health equity professionals?”
- Qualitative: “What support components are described by health equity professionals who report receiving a high level of support in their workplaces?”

**Fig. 1:**
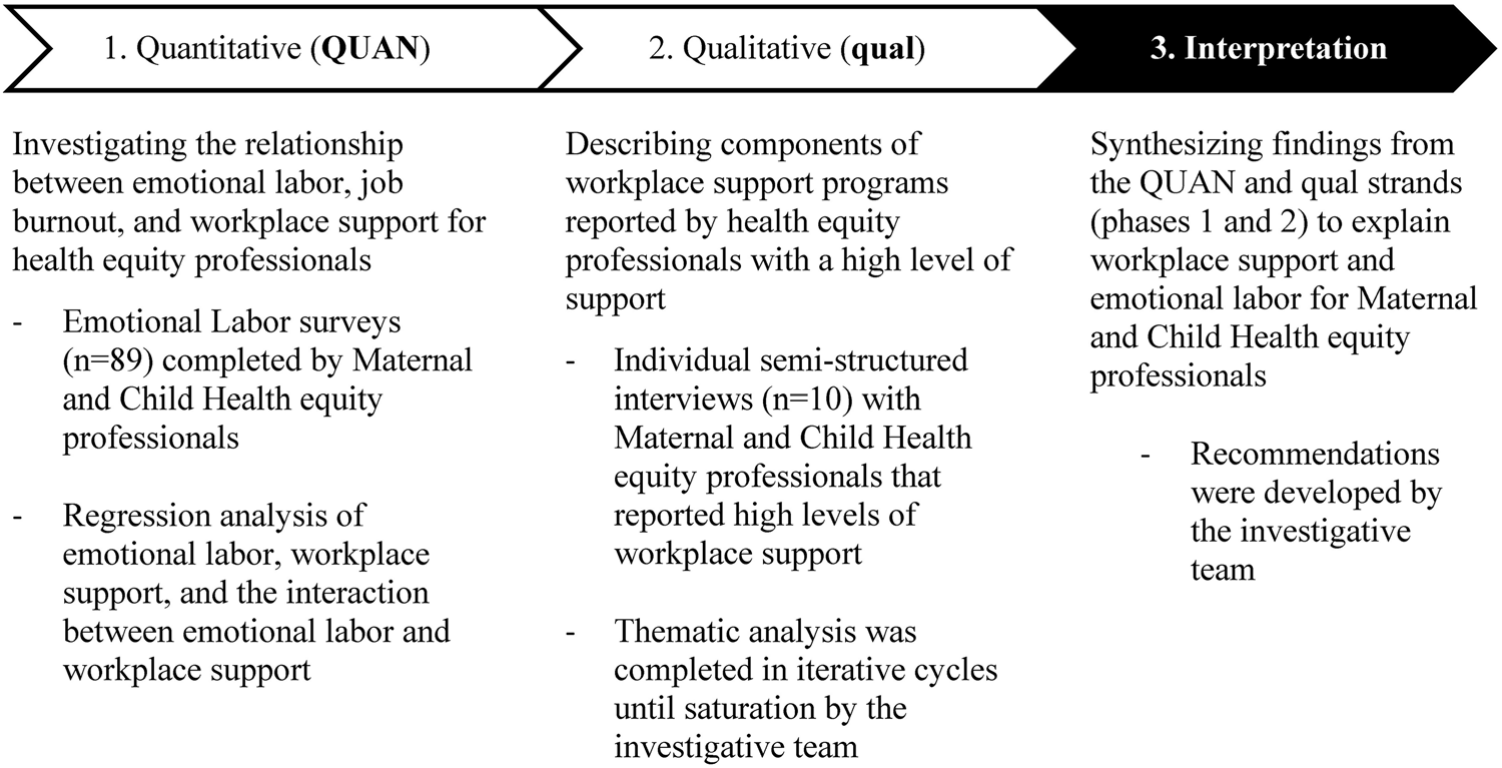
Overview of the mixed-methods explanatory, sequential study design

To address these questions, we conducted a national mixed-methods study (Creswell & Clark, 2017) of public health equity professionals, focusing on the field of MCH because of its emphasis on equity work. Participants were recruited using a snowball sampling approach (Parker et al., 2019) beginning with a seed list of members of three national MCH organizations representing state and local health departments, and community-based MCH programs. Survey topics included emotional labor, burnout, and supports (Abresch et al., 2022). Following a sequential, explanatory study (QUAN-qual) design, a subset of survey respondents was interviewed (Fig. 1).

The survey was fielded, and interviews were completed between August 2020 and June 2021 via Zoom. The study was approved by the lead researchers’ Institutional Review Board (IRB# 628-19-EX).

### Analytic Procedures

The survey tool was adapted from a validated instrument, the GNM Emotional Labor Questionnaire (Guy et al., 2014), and was described in a previous paper (Abresch et al., 2022). For this study, survey participants who provided complete responses were grouped according to their answer to the single question, “I receive the emotional support I need from my employer to perform health equity work.” Response options were on a seven-point Likert scale (i.e., “never,” “rarely,” “occasionally,” “some of the time,” “frequently,” “usually,” or “all the time”). Those reporting that they received this support “frequently,” “usually,” or “all the time” were classified as receiving a high level of workplace support. All other responses were classified as receiving a low level of workplace support.

#### Phase One: Quantitative Analyses

The difference in burnout between those with high and low workplace support was analyzed using the Mann–Whitney *U* Test. In the regression model, the dependent variable was job burnout, which was measured as the average score of four items. Emotional labor, measured as the average of five items, was the independent variable. The dichotomized workplace support variable was in the model as an effect modifier. Scales and items are listed in Table 1. As a secondary analysis, we adjusted the model for multiple covariates (age, race, gender, years of work experience, and organizational size). Normality of residuals was verified using a normal plot and the Anderson-Darling test. Analyses were completed using SAS v 9.4.

**Table 1:** Survey scales and individual items

Dependent variable: Burnout
Health equity work leaves me feeling emotionally exhausted
Health equity work puts a lot of stress on me
Health equity work makes me feel used up.
I worry that health equity work is hardening me emotionally
Independent variable: Emotional labor
Health equity work produces many different emotions for me
Health equity work requires me to guide others through sensitive and/or emotional issues
My health equity work involves dealing with emotionally charged issues as a critical dimension of the job
Health equity work requires me to deal with unpleasant issues
I need to shield myself from feeling the emotions involved in health equity work
Effect modifier: Workplace support
I receive the emotional support I need from my employer to perform health equity work

*Note*: Each response was on a seven-point Likert scale

#### Phase Two: Qualitative Analyses

We purposively selected interview transcripts from participants reporting a high level of workplace support and applied open coding to discover specific components of their support systems deemed important by the interviewees. Rather than seeking to quantify important components across multiple participants, we sought to identify all workplace support components present.

Workplace support components were triangulated among the investigative team based on shared analysis of the emergent themes and supporting participant quotes. We applied the principle of consensus to transcript coding, requiring all investigators to agree on the presence of a theme for inclusion. Direct participant quotes were used to illustrate and, where possible, name the workplace support components.

## Assessment

### Quantitative Findings

Detailed demographics for 91 respondents were published previously (Abresch et al., 2022). Of the 89 respondents with complete data for this study, 52% were age 45 or older and 48% had been working in health equity for more than 10 years, 88% were female, and 53% were Black. Employment sectors included nonprofit/community-based (31%), local public health (26%), state public health (22%), healthcare (12%), education (6%), tribal (1%), and federal (1%). The emotional labor score averaged 5.6 and job burnout averaged 4.1. These factors were positively associated, with a Pearson correlation coefficient of 0.63 (p < .001). Sixty-one respondents (69%) were classified as having low workplace support. Among these, the average burnout score was 5.6 and burnout was positively correlated with emotional labor (0.73, p < .0001). The remaining 28 respondents, with high workplace support, had a lower average burnout score of 3.5 and a reduced correlation between emotional labor and job burnout (0.54, p = .003). The difference in burnout score between workplace support groups (2.1 units) was statistically significant (p = .002) and meaningful, at roughly the difference between a neutral response (score = 4) and agreement (score = 6) on a burnout item.

The statistically significant coefficient for emotional labor in the regression model (Table 2) means that, holding workplace support constant, a unit increase in emotional labor increases predicted job burnout by 1.38 units. The negative interaction term means that the effect of emotional labor on job burnout is significantly decreased in the presence of workplace support. Residuals were normally distributed (p = .150). A likelihood ratio test indicates that adding the two workplace support terms significantly improves model fit (p = .016). The secondary, adjusted analysis, found two significant associations, age and years of work experience, neither of which changed the effect size or significance of the interaction between workplace support and emotional labor.

**Table 2:** Regression analysis

Parameter	DF	Estimate	Wald 95% confidence limits		Wald Chi-square	p-value
Intercept	1	−3.37	−5.33	−1.41	11.39	0.0007
Emotional Labor (EL)	1	1.38	1.03	1.72	61.25	< .001
Workplace Support (WS)	1	2.38	−0.67	5.43	2.33	0.126
Interaction (EL * WS)	1	0.58	−1.11	−0.04	4.39	0.036

*DF* Degrees of Freedom,* EL* Emotional Labor,* WS* Workplace Support

**Fig. 2:**
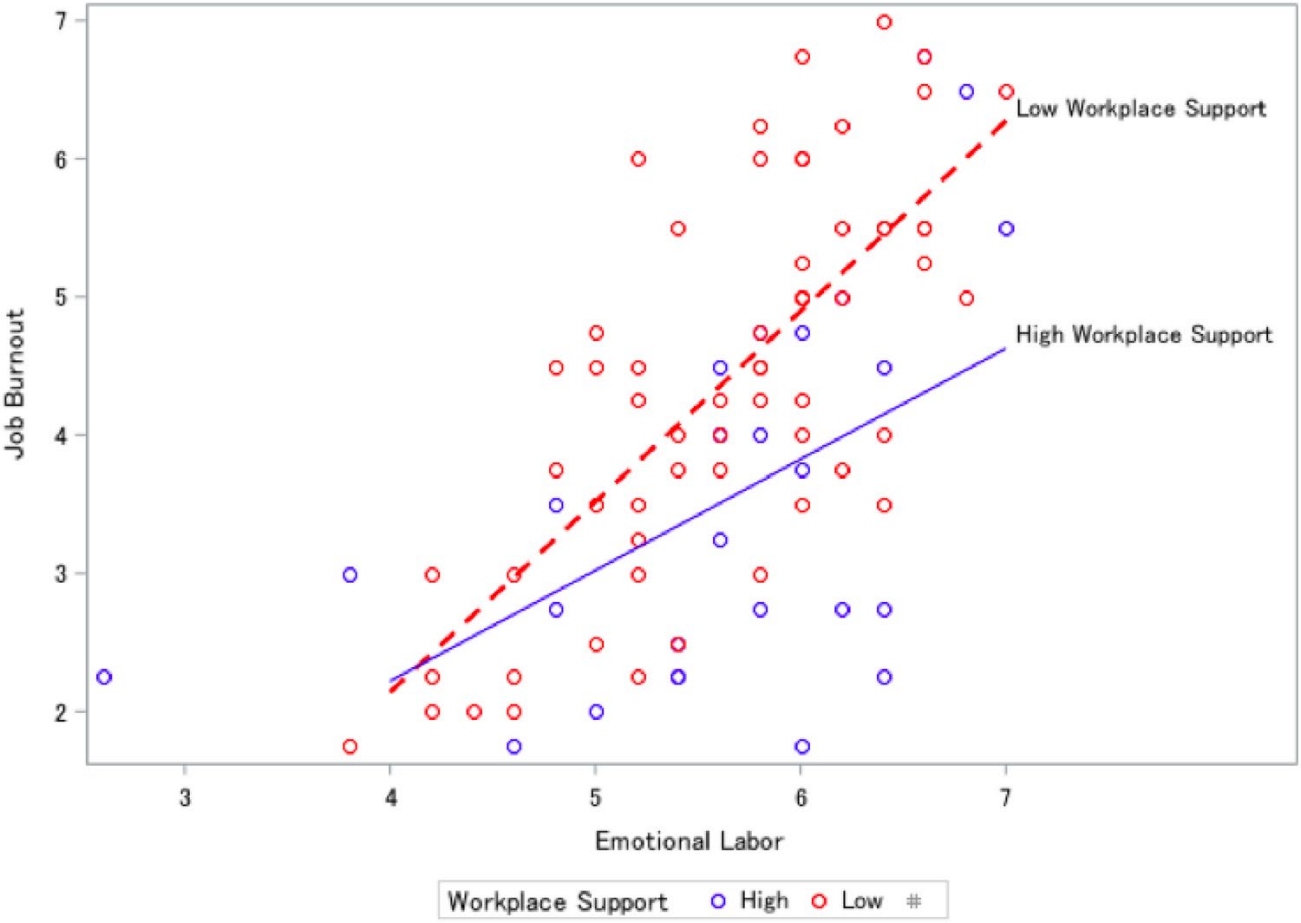
Low vs. high workplace support and level of burnout by the amount of emotional labor reported

Figure 2 shows data points and predicted regression lines for respondents with low workplace support in red, and those with high workplace support in blue. In general, higher emotional labor scores predict higher job burnout scores, but the steeper slope of the red dashed line shows the greater effect of emotional labor on job burnout among people reporting low workplace support.

### Qualitative Findings

Interviews were conducted on a subset of 19 survey respondents who were purposively selected until saturation was reached (Abresch e al., 2022). Analysis of the ten interviewees who were classified as having high workplace support identified four components of workplace support: (1) peer-to-peer mentoring connections, (2) workplace accommodation, (3) engaged and empathetic supervision, and (4) mental health resources.

#### Peer-to-Peer Mentoring Connections

Participants discussed the importance of having time and support for peer-to-peer mentoring connections with like-minded health equity professionals. The value of these connections was described as providing opportunities for candid communication, exchange of ideas, providing mutual encouragement, and simply knowing that others were engaged in this challenging work. One interviewee said, “We put together a group of people to have a candid space to talk about equity issues… that’s both within the health equity team and within the health department. It really feels like kindred spirits. The people who get it. I don’t have to explain it. The people who are invested in my growth and the growth of the health department.”

#### Workplace Accommodations

A second component of support stressed by participants was supportive accommodations made within workplaces. These accommodations included policies that foster a culture of mutual support, including workplaces making time during the workday to connect informally, and allowing for unplanned time off when job demands became too intense. A respondent explained that in their workplace, “We do some things to acknowledge the emotional toll of doing this work, and we’ve agreed to that as a team… If someone just needs to take the day off because it’s all too much, they don’t have to look for it to be the perfect day. They can just take the day. The team has agreed to cover. It doesn’t matter what you have on your calendar that day when you take the day.”

#### Engaged and Empathetic Leadership

A third component of workplace support involved having a leader or supervisor who understands and is willing to invest the time to listen and support health equity professionals. This theme surfaced repeatedly as a key component in the ability to deal with the emotional demands of equity work. One respondent described this kind of support: “My boss is someone that I can talk to. He’s a little bit older than me. And he’s honestly been in the public health arena for a lot longer than I have. And he understands city politics and the game, if you will, he understands that very well. I’m fortunate that I have him to go to when things do get challenging, you know, because they do. So, it is nice to sort of have that, you know, that calm voice, who can just really educate me, during those times when I’m on the edge, and I need to be talked down… Definitely helps in the health equity arena.”

#### Mental Health Resources

The fourth component of workplace support noted was availability of mental health resources provided to meet the demands of health equity professionals. Many employers offer such resources for their employees as part of benefits packages, and interviewee comments in our study validated these offerings. One interviewee was emphatic about feeling supported in this regard: “My job offers resources. Absolutely. For us, you know, they offer access to counseling, that we as employees, we don’t have to pay for.”

In addition to the availability of professional counseling services, other respondents discussed the value of supplemental and tailored mental wellness activities. As an example, one interviewee noted: “I think in the workplace we also have a really great mental wellness team that has implemented some healing circles and other kinds of activities and discussion forums.”

## Conclusion

This study is the first to examine the relationship among emotional labor, job burnout, and workplace support for health equity professionals. The quantitative results indicate that the association between emotional labor and job burnout in this population was weaker when workplace support was high. The qualitative results illustrate ways that workplaces can provide support for the emotional labor demands of health equity work. Taken together, these findings suggest that it is possible to provide workplace supports that could reduce burnout. Our study suggests four components are important in providing meaningful workplace support for the emotional labor of health equity professionals.

1. Peer-to-peer mentoring connections should be established with equity professionals within and/or outside the organization. These relationships appear to ease the emotional burden by providing an outlet to exchange ideas, share frustrations, and connect with like-minded colleagues.
2. The demands of public health equity work require workplace accommodations. Interviewees suggested that the emotional burdens associated with the work were eased when an intentional supportive atmosphere was created, or when they could take time off with their responsibilities covered by other members of the team.
3. Public health equity workers benefit from engaged and empathetic leadership. Ideally, the leadership would know the community and its politics well, understand the emotional difficulty of equity work, and be willing to check in regularly with their employees.
4. Workplaces that employ health equity professionals should ensure the availability of mental health resources. These resources will include the standard array of employee assistance and counseling services and possibly extend to include unique team-building offerings centered on the mental well-being of their equity staff.

Previous studies found that workplace support or supportive leadership can reduce burnout and emotional labor in other settings (Huang et al., 2015; Jeung & Chang, 2021; Yeh et al., 2020). A survey of public health workers documented increasing turnover with lack of workplace support as a driver (de Beaumont Foundation, 2017). Maslach and Leiter found that workplace civility, including enhancing a teamwork perspective, led to enduring reductions in burnout (Maslach & Leiter, 2017). Our findings in public health equity work settings are consistent with these.

Our study has limitations that should be considered when interpreting the findings. First, we used a snowball and convenience sample of individuals engaged in health equity work in the public health subfield of MCH, which limits generalizability and may affect the statistical independence of subjects. Second, although controlling for basic covariates did not significantly alter quantitative findings, we likely did not measure all important confounders. Thus, this study should be viewed as an initial foray into the topic, with additional research needed to replicate and generalize findings. Third, inclusion in our study was based on self-identification as a public health equity worker, chiefly in MCH. We did not collect details on the types of public health equity work our participants performed. It is likely that their day-to-day activities varied widely. Future research is needed to characterize “equity work” and determine the relevance and legitimacy of identifying the subpopulation of “equity workers” in the field of public health.

### Implications for Policy & Practice

Our study suggests that more could and should be done within public health agencies to support health equity professionals who face emotional labor demands and prevent job burnout. These findings align with studies that call for reducing turnover by improving the workplace environment in public health agencies (Harper et al., 2015; Liss-Levinson et al., 2015; Yeager et al., 2016). Public Health Accreditation Board standard, Measure 8.2.3, calls for comprehensive policies by public health agencies to build a supportive work environment (Public Health Accreditation Board, 2022), and our findings provide needed guidance on what supports are helpful. Additional research on the implementation of workplace supports and their effectiveness in addressing the emotional labor, burnout, and retention of health equity professionals are important next steps.

## Data Availability

Quantitative data utilized for analysis in this manuscript is available upon request. Qualitative data is not available on request due to a potential loss of confidentiality for participants.
